# Evolution of the canonical sex chromosomes of the guppy and its relatives

**DOI:** 10.1093/g3journal/jkab435

**Published:** 2021-12-21

**Authors:** Mark Kirkpatrick, Jason M Sardell, Brendan J Pinto, Groves Dixon, Catherine L Peichel, Manfred Schartl

**Affiliations:** 1 Department of Integrative Biology, University of Texas, Austin, TX 78712, USA; 2 Milwaukee Public Museum, Milwaukee, WI 53233, USA; 3 Institute of Ecology and Evolution, University of Bern, Bern 3012, Switzerland; 4 Developmental Biochemistry, University of Würzburg, Würzburg97074, Germany; 5 Department of Chemistry and Biochemistry, The Xiphophorus Genetic Stock Center, Texas State University, San Marcos, TX 78666, USA

**Keywords:** sex chromosomes, recombination, sex determination, heterochiasmy, X chromosome, Y chromosome

## Abstract

The sex chromosomes of the guppy, *Poecilia reticulata*, and its close relatives are of particular interest: they are much younger than the highly degenerate sex chromosomes of model systems such as humans and *Drosophila melanogaster*, and they carry many of the genes responsible for the males’ dramatic coloration. Over the last decade, several studies have analyzed these sex chromosomes using a variety of approaches including sequencing genomes and transcriptomes, cytology, and linkage mapping. Conflicting conclusions have emerged, in particular concerning the history of the sex chromosomes and the evolution of suppressed recombination between the X and Y. Here, we address these controversies by reviewing the evidence and reanalyzing data. We find no evidence of a nonrecombining sex-determining region or evolutionary strata in *P. reticulata.* Furthermore, we find that the data most strongly support the hypothesis that the sex-determining regions of 2 close relatives of the guppy, *Poecilia wingei* and *Micropoecilia picta*, evolved independently after their lineages diverged. We identify possible causes of conflicting results in previous studies and suggest best practices going forward.

## Introduction

The origin and evolution of young sex chromosomes are of particular interest to evolutionary genomics. They are the most rapidly evolving part of the genome in many animals and plants, and they have features that give unique insights into the evolution of recombination, sexually antagonistic selection, and other important processes (Bachtrog et al. 2011). The guppy, *Poecilia reticulata*, holds a special place in the history of this subject. Because they carry many of the genes responsible for the males’ famed coloration, the *Poeciliidae reticulata* sex chromosomes have been studied since the 1920s (Winge 1922; Haskins et al. 1961; Lindholm and Breden 2002; Charlesworth 2018). The last decade has seen a burst of research on the sex chromosomes of the guppy and its relatives. Several recent studies have arrived at conflicting conclusions, notably regarding the evolution of recombination. These controversies are the focus of this study.

Studies from the pregenomic era provided conflicting conclusions regarding recombination between the sex chromosomes. Using linkage maps, Tripathi et al. (2009) reported that recombination between the X and Y of *P. reticulata* is confined to a relatively small region bounded on either side by large nonrecombining regions. Nanda et al. (2014) used cytology and linkage maps to study the sex chromosomes of *P. reticulata*, its sister species *Poeciliawingei*, and the closely related *Poeciliaobscura*. They concluded that the Y chromosomes of these 3 species descended from a common ancestral Y, and that the X and Y chromosomes of *P. reticulata* recombine down their lengths (save perhaps for a small heterochromatic region specific to the Y). Further, they reported that an extended region of heterochromatin at the end of the Y distal to the centromere has evolved in *P.wingei* that would regionally block recombination with the X. Conversely, by inferring meiotic crossovers visualized through the localization of MHL1, Lisachov et al. (2015) found that recombination between the X and Y in *P. reticulata* is concentrated toward the end of the chromosome distal to the centromere.

The pace of discovery accelerated with the arrival of genome sequences for *P. reticulata* (Fraser et al. 2015; Künstner et al. 2016). By analyzing molecular variation among re-sequenced genomes from several natural populations of *P. reticulata*, Wright et al. (2017) drew conclusions at odds with the previous reports. They found that crossing over between the X and Y is completely blocked over about 40% (10 Mb) of their lengths, with recombining regions on either side. They claimed that the nonrecombining sex-determining region (SDR), is divided into 2 “strata,” which are generally defined as regions in which crossing over between the X and Y was completely suppressed at different times in the past (Lahn and Page 1999; Charlesworth 2017). Wright et al. (2017) further concluded that the nonrecombining SDR has expanded independently in 3 populations that inhabit the headwaters of rainforest streams. Morris et al. (2018) followed up this study by identifying 40 loci that are unique to the putative nonrecombining region of the Y chromosome, and proposed 2 of them as candidates for the sex-determining gene.

In the next study from the same research team, Darolti et al. (2019) enlarged the phylogenetic picture by analyzing genomes and transcriptomes from 5 species of poeciliid fish, including *P. reticulata*, its sister species *P. wingei*, and the closely related *Micropoecilia picta* (formerly called *Poecilia picta*). These authors found that chromosome 12 is responsible for sex determination in all 3 species. They reported that the 2 strata that they found on the Y of *P. reticulata* are shared with *P. wingei*, which implies that they evolved in the common ancestor of those species. In the more distantly related *M. picta*, they found the Y chromosome to be highly degenerate. They also concluded based on parsimony that the SDRs in all 3 species descend from a common ancestor, which implies that the rates at which the Y degenerates varies greatly between lineages. Darolti et al. (2020) concluded from linkage mapping that completely suppressed recombination is confined only to the first of the 2 strata in *P. reticulata*, while there is very rare crossing over between the X and Y in the second “stratum.” In a more recent study from that research group, Almeida et al. (2021) carried out long-read sequencing on much larger samples of individuals from 6 natural populations. They again concluded that the data show that there is no recombination in Stratum 1.

An independent research team studied the *P. reticulata* sex chromosomes using linkage mapping (Bergero et al. 2019). They found no evidence for completely suppressed recombination between the X and Y anywhere on those chromosomes. Recombination between the X and Y in natural populations must be very rare, however, as there is elevated *F*_ST_ between males and females down the entire length of chromosome 12, in some regions attaining values greater than 0.1. Bergero et al. (2019) showed that the very low levels of recombination between the X and Y are not unique to the sex chromosomes: males also have extremely low recombination rates on autosomes except near the telomeres. They also identified a region of high recombination on the end of the Y chromosome distal to the centromere, consistent with the patterns of male recombination on autosomes and the findings of Lisachov et al. (2015). The localization of crossovers in males to the tips of all chromosomes is consistent with the high GC content found there, since GC content is correlated with rates of recombination (Charlesworth et al. 2020b). Charlesworth et al. (2020a) also reported a crossover between the X and Y of *P. reticulata* very close to the boundaries between strata 1 and 2 as defined Almeida et al. (2021).

Fraser et al. (2020) assembled a new high-quality reference genome for *P. reticulata* that includes unphased scaffolds from both the X and Y, and they re-sequenced fish from 6 natural populations. Those authors concluded that if an SDR exists, it is very small (<1 Mb). They found 2 candidate regions for the sex-determining gene contained in Y-specific regions that (surprisingly) are located at opposite ends of the Y chromosome. The sequence of the most likely candidate is absent from the X chromosome. This would explain why it was not detected in other studies that are based on earlier reference genomes that lack Y-specific sequences. They concurred with Bergero et al. (2019) that there is no evidence for a nonrecombining stratum several megabases in size.

**Fig. 1.**
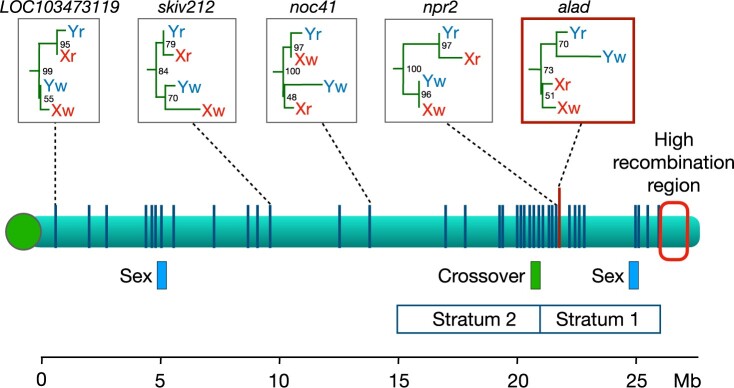
The sex chromosome of the guppy, *P. reticulata*. The green bar shows the interval in which a crossover was observed by Bergero et al. (2019) and Charlesworth D (personal communication). Strata 1 and 2 are regions where Wright et al. (2017) reported that the X and Y do not recombine. Blue boxes labeled “Sex” are candidate regions for the male-determining factor (Fraser et al. 2020). At far right is a region with a high local recombination rate (Lisachov et al. 2015; Bergero et al. 2019; Darolti et al. 2019). Vertical blue lines show locations of the 42 loci at which gene trees from *P. reticulata* (Xr, Yr) and *P. wingei* (Xw, Yw) were estimated by Darolti et al. (2020). Only the gene tree highlighted in the red box has a topology consistent with an ancestral SDR shared by the 2 species; examples of 4 other representative gene trees are also shown. Numbers at their nodes give the bootstrap support. The centromere is shown as the green circle at left.

Figure 1 summarizes some of these previous results. Very recently, Charlesworth et al. (2021) analyzed sequence data from *M. picta* and the closely related *Micropoeciliaparae*. Both species have highly degenerate Y chromosomes, and 99% of genes have been lost from the SDR on the Y of *M. picta*. They conclude that their SDRs are not homologous with that of the guppy because there has not been enough time since the 2 lineages diverged for such extreme degeneration to accumulate in the Y of the *M. picta*. They support the alternative hypothesis that the guppy lineage recently underwent a turnover event in which a new and undegenerated Y was derived from its X chromosome.

These conflicting conclusions have led to controversy and confusion in the scientific community (Bergero and Charlesworth 2019; Wright et al. 2019). In an effort to remedy this situation, in this study, we review, reanalyze, and reinterpret previously published data from the guppy and its close relatives. Our goal is not to perform a forensic analysis to determine why different research teams came to different conclusions. Rather, we analyze the existing data in order to independently evaluate conclusions regarding the evolutionary histories of their sex chromosomes.

We first focus on the evolution of recombination and the origin of the Y chromosomes. We find no evidence for strata in *P. reticulata* or for any region where crossing over between the X and Y is completely suppressed. Our results confirm that nonrecombining SDRs span much of the X and Y in *P. wingei* and *M. picta*, and that the Y in *M. picta* is highly degenerate.

We then consider hypotheses regarding homology of the SDRs in all 3 species. By “homology,” we mean that the SDRs in these species descended from a common ancestral SDR. While the shared identity of the Y chromosomes of *P. reticulata* and *P. wingei* has been established by molecular cytogenetics (Nanda et al. 2014), that approach cannot evaluate the homology of their SDRs. Our analysis of patterns of allele sharing does not support the hypothesis that the SDRs of *P. wingei* and *M. picta* descend from a common ancestral SDR. A second hypothesis is that the Y chromosomes of *P. wingei* and *P. reticulata* originated in their common ancestor in a turnover event after divergence from the *M. picta* lineage (Bergero and Charlesworth 2019; Meisel 2020; Charlesworth et al. 2021). This would make their Y chromosomes younger, and so explain why they are so much less degenerate than the Y of *M. picta*. We find the hypothesis most strongly supported by patterns of allele sharing is that the SDRs of *P. wingei* and *M. picta* originated independently. This conclusion is consistent with the turnover hypothesis, but it is also consistent with the independent suppression of recombination after the 2 lineages diverged.

## Materials and methods

We downloaded all of the whole genome re-sequencing data available as of June 2020 from the 2 Bioprojects hosted on the NCBI SRA database ([dataset]* University College London, 2016, Recent origin and rapid spread of recombination suppression between the guppy X and Y chromosomes suggests a role for sexual selection in sex chromosome formation, NCBI Short Read Archive, PRJNA353986; [dataset]* University College London, 2019, Extreme heterogeneity in sex chromosome differentiation and dosage compensation in guppies, NCBI Short Read Archive, PRJNA528814). These data were collected by Wright et al. (2017) and Darolti et al. (2019). [We did not use the more recent data from Fraser et al. (2020) or Almeida et al. (2021) for most of our analyses because they were published after our study was first submitted]. The data consist of paired-end Illumina sequences from 52 samples of whole-genome sequences of 5 species: *P.reticulata*, *P. wingei*, *P. latipinna*, *M.picta*, and *Gambusia holbrooki*. Our main focus was on the samples from *P. reticulata* (2 females and 26 males), *P. wingei* (3 females and 3 males), and *M. picta* (3 females and 3 males). For a given data type, multiple records from the same individual were concatenated. Read quality was assessed using FastQC [v0.11.5] (Andrews 2010).

For most analyses, we mapped WGS reads to the *Xiphophorus maculatus* reference genome [v5.0; GCA_002775205.2; Schartl et al. (2013)] because it is the highest-quality poeciliid fish genome and is an equal phylogenetic distance from our 3 focal species. For the analyses of allele sharing (see below), we redid our original analyses using the new *P. reticulata* reference genome (GCA_904066995.1), which was published after our study was first submitted. We used this second genome with the goal of increasing read mapping rates across poeciliid species. Indeed, we found that the mapping efficiency with sequencing reads from *P. reticulata* is more than twice as high when using the new *P. reticulata* reference (∼93%) as when using the *X. maculatus* reference (∼41%), and it was similar across all 3 focal taxa. However, there are no differences in read mapping efficiency between males and females using either of the 2 reference genomes, which makes *X. maculatus* an appropriate reference for the other analyses. There are small differences in the analysis pipelines using the *X. maculatus* and *P. reticulata* reference genomes that are specified below.

There is some uncertainty about the physical coordinates on the *P. reticulata* sex chromosomes. Different studies have used different reference genomes that vary in important features [e.g. Wright et al. (2017) vs Bergero et al. (2019) vs Fraser et al. (2020) vs this study]. Furthermore, there are several reports of inversions and assembly errors on the *P. reticulata* sex chromosomes (Nanda et al. 2014; Bergero et al. 2019; Darolti et al. 2020; Charlesworth et al. 2020a; Fraser et al. 2020). Accordingly, we interpret the chromosome coordinates with caution.

### Analyses based on the *X. maculatus* genome

We trimmed adapters and low-quality bases from raw SRA sequence reads using cutadapt [v1.14] (Martin 2011), mapped DNA and RNA reads to the *X. maculatus* reference genome using Bowtie2 [v2.3.4] with default parameters and the –local argument (Langmead and Salzberg 2012). We removed PCR duplicates using Picard [v2.21] (Broad Institute 2019). We sorted and subsetted alignment files by chromosome using SAMtools [v1.6] (Li et al. 2009) and calculated fold coverage in 10 kb windows using BEDtools [v2.26.0] (Quinlan and Hall 2010). We called variants with a quality score ≥ 20 using BCFtools mpileup [v1.10] (Li 2011). We removed indels and singletons, selected for biallelic SNPs using VCFtools [v0.1.16] (Danecek et al. 2011), and we required a minimum read depth of 3.

To determine which SNPs may be in an SDR, we considered patterns of heterozygosity. An allele was considered to be putatively Y-linked if it always appeared in males in heterozygotes and was absent from females. We designate these alleles as “Y-like,” the alternate alleles at these SNPs as “X-like,” and the sites at which these occur as “SDR-like SNPs.” We imposed the additional criterion that these sites have data from at least 2 individuals of each sex. Because the sample sizes are small, SDR-like SNPs can occur by chance at autosomal loci. Nevertheless, this set of sites will be highly enriched for SNPs that truly are sex-linked. As an internal control, we also applied this analysis to the autosomes to assess how frequently SNPs with putative sex linkage occur throughout the genome.

To study divergence between the X and Y chromosomes, we computed 5 statistics in sliding windows: (1) the ratio of read depth in males and females (“read depth ratio”), computed using BEDtools [v2.26.0] (Quinlan and Hall 2010); (2) *F*_ST_ between males and females (“*F*_ST_”), based on Weir and Cockerham’s (1984) estimator computed using VCFtools [v0.1.16] (Danecek et al. 2011); (3) the ratio of the density of all SNPs in males and in females (“SNP density ratio”), computed using VCFtools and a custom R script; (4) the density of SNPs with patterns of heterozygosity consistent with the SDR (“SDR-like SNP density”), computed using VCFtools and a custom R script; and (5) the density of SNPs with alleles that are restricted to females (“female-specific SNP density”), computed using VCFtools and a custom R script. We examined read depth differences between males and females in 10-kb windows and all other sliding-window statistics in 100-kb windows.

The custom scripts used for data processing, analysis, and figure generation based on the *X. maculatus* genome are available at https://github.com/grovesdixon/guppy_sex_chroms (DOI: https://zenodo.org/badge/latestdoi/239392218). The SNPs we called are available at DOI: 10.6084/m9.figshare.17203502.

### Analyses based on the *P. reticulata* genome

We quality and adapter trimmed the sequencing reads using Trim Galore! [v0.6.6] (Andrews 2010; Martin 2011), filtered PCR duplicates using bbmap [v38.90] (Bushnell 2014), and mapped reads to the *P. reticulata* reference genome using minimap2 [2.17] (Li 2018). We called variants one chromosome at a time at sites with quality score ≥20 using BCFtools mpileup [v1.11] (Li 2011). We removed indels and singletons, selected for biallelic SNPs using VCFtools [v0.1.16] (Danecek et al. 2011), and we required a minimum read depth of 10. Scripts for the QC and read mapping to the *P. reticulata* genome have been previously published (Pinto et al. 2021).

We implemented this strategy using a custom R script. We first split the genotypes by species using VCFtools. We then filtered SNPs to include only those with no missing individuals (–max-missing 1.0) and a minimum allele frequency of 0.2 (–maf 0.2). We then split the genotypes further by sex and calculated allele frequencies (–freq) and observed heterozygosity (–hardy) in VCFtools.

We used patterns of allele sharing at SNPs to test hypotheses regarding the evolution of suppressed recombination. We applied several filters with a custom R script to identify the SNPs to use. We refer to the SNPs that meet all of the following criteria as “topologically informative.” First, we required sequence from at least 2 *P. wingei* males, 2 *P. wingei* females, 2 *M. picta* males, 2 *M. picta* females, 1 *P. latipinna*, and 1 *G. holbrooki*. Second, we only considered SNPs where alleles are fixed on the X or on the Y chromosomes for *P. wingei* and *M. picta*. That is, we required that the females within a species all be homozygous for the same allele, and that the males in that species either be all heterozygous or all homozygous for the same allele found in females. Third, we required that all individuals from the outgroup species (*P. latipinna* and *G. holbrooki*) possess the same allele, which we identified as “ancestral.” Finally, we required that the alternate (derived) allele be fixed in either 2 or 3 of the following 4 chromosomes: the *P. wingei* X, *P. wingei* Y, *M. picta* X, or *M. picta* Y. We used the same R script to calculate the number of topologically informative SNPs that are consistent with each of the 10 possible gene trees shown in Fig. 5. The custom R scripts used for these analyses are available at https://github.com/JasonSardell/Poecilia-gene-tree-scripts.

## Results and discussion

### Divergence and recombination between the X and Y

#### The X and Y of guppies

**Fig. 2.**
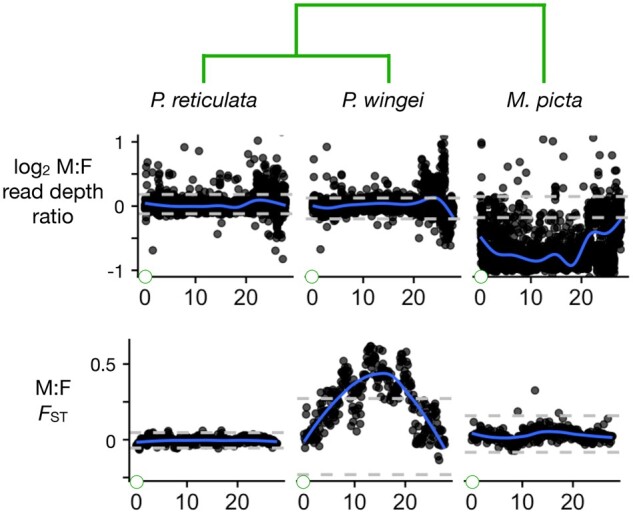
Divergence between the X and Y chromosomes in the focal species. Two statistics that measure differences between the sexes as a proxy for differences between the X and Y: the male: female read depth ratio, and the male: female *F*_ST_. The gray horizontal dashed lines show the bottom 2.5% and top 97.5% intervals based on windows from all autosomes. The blue curves are smoothed regressions. Green circles at the left of the *Y*-axes represent the centromere. The phylogeny is shown at top.

**Fig. 3.**
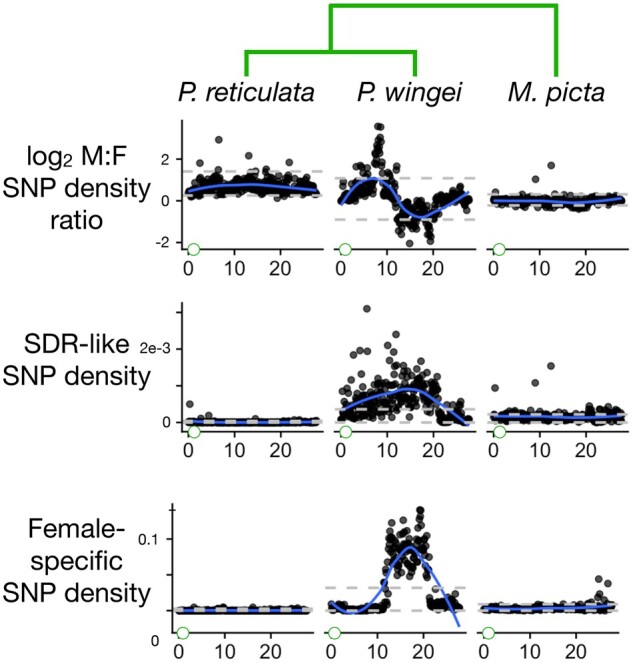
Divergence between the X and Y chromosomes as measured by 3 additional statistics. These are the male: female SNP density ratio, the SDR-like SNP density, and the female-specific SNP density. The gray horizontal dashed lines show the bottom 2.5% and top 97.5% intervals based on windows from all autosomes. The blue curves are smoothed regressions. Green circles at the left of the *Y*-axes represent the centromere. The phylogeny is shown at top.

Five lines of evidence argue against the existence of nonrecombining strata on the sex chromosomes of *P. reticulata*. The first is based on several summary statistics that measure differences between males and females on the sex chromosomes (a proxy for differences between X and Y). We examined these statistics in sliding windows using the syntenic linkage group 8 of *X. maculatus* as the reference. Figure 2 shows the read depth ratio and male: female *F*_ST_, while Fig. 3 shows the male: female SNP density ratio, SDR-like SNP density, and female-specific SNP density. Supplementary Fig. 1 shows the densities of SDR-like SNPs on the sex chromosomes and autosomes. As anticipated, SDR-like SNPs do occur on autosomes, but are much less common there (see *Materials andMethods*).

In *P. reticulata*, there is no sign that the X and Y have diverged very much anywhere along their lengths. We do not see a decreased read depth ratio, which is a telltale signature of Y chromosome degeneration (Vicoso and Bachtrog 2015). The maximum value of the smoothed regression for *F*_ST_ between males and females is less than 0.003 down the entire length of the sex chromosomes (Fig. 2). The maximum value in any 100 kb window is *F*_ST_ = 0.15. We obtained similar results for *F*_ST_ using both the *P. reticulata* and the *X. maculatus* reference genomes. The other 4 statistics fall within the ranges typical for autosomes. These results do not provide any evidence of a nonrecombining SDR.

Second, published gene trees likewise fail to show evidence of strata on the guppy sex chromosomes. Following the origin of a nonrecombining stratum on the Y by an inversion (or any other mechanism involving a *cis* recombination modifier), the SDRs on all Y chromosomes that inherit that stratum will form a monophyletic group with respect to the X chromosomes (Lahn and Page 1999; Zhou et al. 2014). The monophyly persists through speciation events: the homologous strata on the Ys from all the descendant species continue to form a clade with respect to the X chromosomes, and the monophyly extends across the entire length of the stratum. This monophyly breaks down, however, in recombining regions of the sex chromosomes. Recombination causes gene copies from the X and Y chromosomes of each species to be intermingled on a gene tree. Recombination events more recent than a speciation event cause gene copies from the X and Y of a species to cluster together rather than with their gametologs in other species. Gene trees thus offer a sensitive way to distinguish the SDR from the recombining pseudoautosomal region (PAR) because they integrate signals of recombination that have accumulated in natural populations over many generations ([Bibr jkab435-B18][Bibr jkab435-B18]; Sardell et al. 2021).

Motivated by that logic, we revisited the gene trees from 2 earlier publications. The results suggest that crossovers have occurred in the region identified as nonrecombining by Wright et al. (2017) and later studies by that research team (Morris et al. 2018; Darolti et al. 2019, 2020; Almeida et al. 2021). Darolti et al. (2020) estimated the gene trees at 42 loci spread along the length of the sex chromosomes. Figure 1 shows their locations. Only one of these loci, *alad*, shows a gene tree topology consistent with a nonrecombining stratum shared between *P. reticulata* and *P. wingei*, but the statistical support for this tree is weak (bootstrap value: 51%). Three other gene trees are also stated to be consistent with that hypothesis, but in fact their topologies are not: see Fig. 2, c–e in Darolti et al. (2020). At the locus *npr2*, which is less than 3 kb away from *alad*, there is strong statistical support for the node joining the X and Y in *P. reticulata* together (bootstrap value: 97%) and for a node joining the X and Y in *P. wingei* (bootstrap value: 96%). This is evidence in both species that there has been recombination between the X and Y chromosomes in the region between the *npr2* locus and the SDR since the 2 species diverged. The remaining 40 gene trees are also inconsistent with a stratum that predates the divergence of *P. reticulata* and *P. wingei*. Most recently, Almeida et al. (2021) analyzed gene trees with much larger samples. In 5 of the 6 populations studied, the majority of trees in the proposed Stratum 1 and elsewhere on chromosome 12 have topologies that are consistent with ongoing recombination between the X and Y in *P. reticulata*. We expect that the most recent recombinant Y chromosome was established within the last few thousand generations, based on the effective population size of Y chromosomes relative to autosomes and the small census population sizes of guppies (Fraser et al. 2015).

A third line of evidence against nonrecombining strata on the guppy sex chromosomes comes from a male crossover that was directly observed in a putative stratum (Bergero et al. 2019; Charlesworth et al. 2020a) (see Fig. 1). It occurred near the 21-Mb position, which is at the approximate boundary between Strata 1 and 2 as defined by Almeida et al. (2021). While Wright et al. (2017) concluded that there is no X–Y crossing over in either stratum, Darolti et al. (2020) do allow for the possibility of rare events in stratum 2.

Fourth, in our analyses, SNPs with heterozygosity patterns consistent with sex linkage are scattered across the length of the sex chromosomes, rather than being restricted to the proposed nonrecombining region (Bergero et al. 2019; Darolti et al. 2020; Almeida et al. 2021).

Finally, Y haplotypes have high nucleotide diversity in guppies (Almeida et al. 2021). This is consistent with the ongoing introduction of genetic variation by recombination from the X to the Y, and is inconsistent with suppressed recombination (no matter how recently it evolved). Almeida et al. propose that the data can be explained if recombination in Stratum 1 was suppressed gradually. But no matter what the mechanism (an inversion, change in chromatin structure, etc.), the fixation of any *cis* modifier that expands the SDR by so much as a single base pair eliminates all variation throughout the entire SDR of the chromosome on which that change occurred. If all the suppression results from inversion(s) on the X, then diversity would be reduced on that chromosome, but that is not what the data show [Fig. 3c in Almeida et al. (2021)]. Variation could be maintained if the SDR expanded—either gradually or rapidly—by one or more unlinked (*trans*) recombination modifiers. We do not know, however, of any species in which that has been observed, nor of any genetic mechanism that has that effect. The high nucleotide diversity on the guppy Y is therefore inconsistent with suppressed recombination, even if it evolved very recently.

Thus, 5 complimentary types of observations strongly argue against the existence of nonrecombining strata on the guppy sex chromosomes. What then can account for the earlier reports of differentiation between the guppy X and Y (Nanda et al. 1992, 1993)? We concur with Bergero et al. (2019) and Charlesworth et al. (2020a), who attribute those observations to the extremely low recombination rates they found on all chromosomes in male guppies. Their findings are consistent with earlier reports in guppies (Tripathi et al. 2009; Lisachov et al. 2015). Apparently, recombination in males is sufficiently rare in at least some populations that the X and Y chromosomes have diverged at the molecular level, e.g. *F*_ST_ between males and females in the range 0.05–0.1 over large regions of the sex chromosomes (Bergero et al. 2019; Almeida et al. 2021). For unknown reasons, our analyses found lower levels of differentiation between males and females, with no clear peaks that exceed values found on the autosomes (Fig. 2).

While the degree of sex bias in recombination (heterochiasmy) is extreme in guppies, qualitatively similar differences between males and females are seen across the eukaryotes (Sardell and Kirkpatrick 2020). In fact, there are examples of heterochiasmy even more extreme than guppies. Recombination along almost the entire lengths of all chromosomes is extremely rare in males of ranid frogs (Brelsford et al. 2016; Jeffries et al. 2018). As a result, *F*_ST_ between males and females approaches its maximum possible value on the sex chromosomes in some populations (Toups et al. 2019).

Variation between populations in recombination rates on the sex chromosomes is a recurring theme in the guppy literature. Using laboratory crosses, Haskins et al. (1961) showed a significant difference between 2 populations in the recombination rate between the sex-linked locus *Sb* and the sex-determining gene. Fraser et al. (2015) reported differences between populations in the linkage of certain color patterns to the X and Y chromosome, but their findings regard differences in the frequencies of color pattern alleles on the X and Y chromosomes rather than differences in recombination rates. In contrast, Wright et al. (2017) and Almeida et al. (2021) argue that differences between populations in patterns of molecular variation reflect differences in the actual rates of recombination between the X and Y.

We suggest that the very subtle differences they reported between populations in the degree of molecular differentiation between the guppy X and Y could occur even in the absence of differences in the recombination landscape. Consider the consequences of a rare crossover between the X and Y, for example near to the male determining gene. If the new Y haplotype spreads to high frequency by selection or drift, differentiation between the X and Y will be erased from the crossover breakpoint down the rest of the chromosome distal to the male determining gene. In the headwaters of the streams, population sizes of *P. reticulata* are only a couple thousand individuals (Fraser et al. 2015). Consequently, crossovers between the X and Y may occur very infrequently, giving the X and Y time to diverge slightly before the next recombinant Y chromosome is established. Downstream populations are many times larger (Fraser et al. 2015). The result will be that recombinant Y chromosomes appear much more frequently in these populations and so divergence between the X and Y has less opportunity to build up.

Differentiation between the guppy X and Y may also result from sexually antagonistic selection, or SAS. More than 50 traits that are under sexual selection in male *P. reticulata* have been mapped to the sex chromosomes (Lindholm and Breden 2002). These are expected to generate peaks in the divergence between the X and Y in neutral genetic variation and can generate patterns that give the appearance of suppressed recombination (Charlesworth 2018; Bergero et al. 2019). Recombination in *P. reticulata* males is so rare that the map length of almost the entire Y chromosome is much less than 1 cM (Bergero et al. 2019). The small census population sizes suggest that many targets of SAS may lie within less than 1 ρ (=*N*_e_*r*/2) of the sex-determining factor, a condition favorable to inflated values of *F*_ST_ between the X and Y (Kirkpatrick and Guerrero 2014). Indeed, peaks in *F*_ST_ between males and females (a proxy for divergence between the X and Y) consistent with SAS have been reported by Bergero et al. (2019) and are visible in Fig. 1 of Almeida et al. (2021). Thus, differences between populations in the intensity of sexual selection and the frequencies of alleles at loci that experience SAS could also contribute to the differences between populations in the degree of X–Y divergence even in the absence of variation in recombination rates.

In summary, we find no evidence for the presence of nonrecombining strata on the guppy sex chromosomes. The subtle differences between guppy populations in the differentiation between X and Y chromosomes could result from variation in population sizes and/or the strength of sexually antagonistic selection.

#### The X and Y of *P. wingei* and *M. picta*

In the sister species of guppies, *P. wingei*, the sex chromosomes are very different. Four of the 5 summary statistics described earlier (all but read depth ratio) fall far outside the autosomal range of values over much of the center part of the sex chromosomes (Figs. 2 and 3). These patterns are consistent with very little or no recombination. The read depth ratio remains near 1, however, suggesting that there has not been extensive degeneration on the Y chromosome, possibly because recombination was suppressed recently. These conclusions concur with Darolti et al. (2019).

Within the region of reduced recombination in *P. wingei*, there is a segment between about 10 and 20 Mb where the female-specific allele density is greatly elevated, with very sharp boundaries at each end (Fig. 3). This pattern in unexpected in species with XY sex determination. The other 3 statistics just mentioned show no unusual patterns specific to this region. We speculate that these patterns result from a polymorphic inversion on the X chromosome of *P. wingei* that is evident in the cytological data of Nanda et al. (2014). The patterns seen in Fig. 3 could result if the 3 X chromosomes in the sample from *P. wingei* males are monomorphic for 1 arrangement while the 6 X chromosomes from females include both arrangements. We are not able to determine if there is more than 1 stratum blocking recombination between the X and Y in *P. wingei*. A segment of the sex chromosomes distal to the centromere shows no sign of molecular divergence between the X and Y, suggesting high rates of recombination there.

*Micropoecilia* *picta* shows yet another distinctive set of patterns. Most strikingly, the read depth ratio is about one half over most of the end of the sex chromosome proximal to the centromere (Fig. 2). This is indicative of large-scale deletions and/or divergence of the Y sequence to the point that reads from it no longer map to the reference (Vicoso 2019). The SDR-like SNP density, female-specific allele density, and *F*_ST_ are much lower than in *P. wingei*, presumably for the same reasons. These conclusions agree with Darolti et al. (2019) and Charlesworth et al. (2021).

### History of the sex chromosomes and their SDRs

**Fig. 4.**
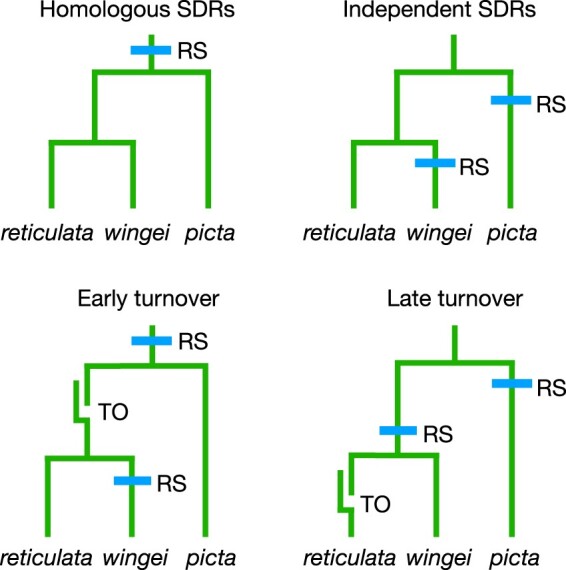
Four hypotheses for the history of the SDRs on the Y chromosomes of the focal species. “RS” = recombination suppression forms an SDR; “TO” = a turnover causes a new Y to be derived from an X. Our analyses reject the homologous SDR hypothesis but cannot decide between the other 3.

We used patterns of alleles shared at SNPs to test competing hypotheses about the evolutionary relations between the SDRs on the Y chromosomes of *P. wingei* and *M. picta*. We do not include *P. reticulata* in these analyses because it lacks a nonrecombining SDR. The hypotheses are shown schematically in Fig. 4.

The first hypothesis is that the oldest parts of their SDRs are homologous, meaning that they descend from an SDR in their common ancestor. This possibility is favored by Darolti et al. (2019), Almeida et al. (2021), and Metzger et al. (2021). The second hypothesis is that the SDRs of *P. wingei* and *M. picta* originated independently, either due to the independent evolution of suppressed recombination of a largely recombining ancestral Y, or independent recruitment of the same autosome as a sex chromosome. The third hypothesis is that the Y chromosome of the shared ancestor of *P. wingei* and *P. reticulata* was derived from its X, an idea favored by Bergero and Charlesworth (2019), Meisel (2020), and Charlesworth et al. (2021). The 4th hypothesis is that a turnover occurred in the guppy lineage after it split from *P. wingei*.

**Fig. 5.**
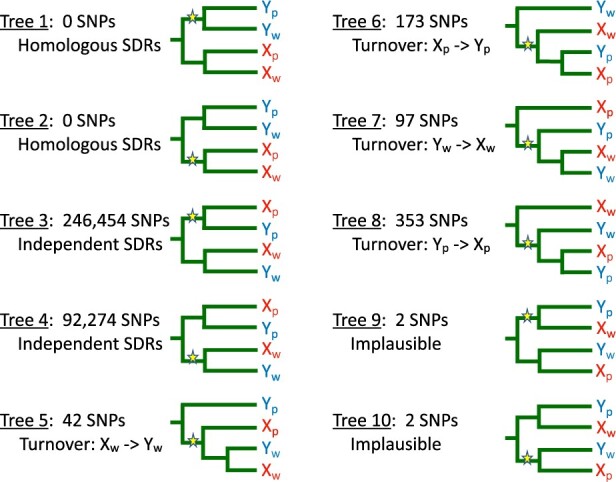
Ten trees for the SDRs of *M. picta* and *P. wingei* inferred from allele sharing patterns. *X_p_* and *Y_p_* represent the sex chromosomes of *M. picta*; *X_w_* and *Y_w_* represent the sex chromosomes of *P. wingei*. Derived mutations are shown by stars. Indicated are the numbers of SNPs at which each tree is seen and a biological interpretation of that tree. All trees can also result from introgression, homoplasy, and sequencing error. Note that the subtrees descending from the mutations shown in trees 5–8 are consistent with the proposed biological scenario, but the allele sharing patterns observed at those SNPs give no information about the evolutionary relations of the 3 types of sex chromosomes that carry the mutant allele. Trees 9 and 10 are biologically implausible and therefore not discussed in the text.

These hypotheses make contrasting predictions regarding the patterns of allele sharing for SNPs in the SDRs. If the SDRs of *P. wingei* and *M. picta* descend from a common ancestral SDR, then the Y chromosomes from both species will cluster together to the exclusion of the X chromosomes (trees 1 and 2 in Fig. 5). If the SDRs evolved independently in the 2 species, then the X and Y of each species will cluster together (trees 3 and 4 in Fig. 5). If the Y of *P. wingei* was derived from its X chromosome after that lineage diverged from *M. picta*, then the X and Y chromosomes of *P. wingei* will cluster together and form a clade that is sister to the *M. picta* X chromosomes (tree 5 in Fig. 5). Other evolutionary histories involving turnover events in which a Y was derived from an X or vice versa result in 3 additional topologies (trees 6–8 in Fig. 5).

None of the 339,397 SNPs on chromosome 12 have either of the patterns of allele sharing expected if the SDRs of *M. picta* and *P. wingei* are homologous (trees 1 and 2). Instead, *M. picta* and *P. wingei* are fixed for different alleles at 99.8% of the SNPs (trees 3 and 4), suggesting that their SDRs evolved independently. Unfortunately, bioinformatic artifacts resulting from Y degeneration can cause these gene trees to be erroneously inferred. At hemizygous sites (where there is a deletion on the Y), BCFtools and other widely used SNP calling software impute a homozygote, which implies the presence of a Y-linked allele that is the same as the X-linked allele. This artifact results in gene trees in which the X and Y chromosomes from *M. picta* appear to cluster together to the exclusion of the X and Y chromosomes of *P. wingei*, which can falsely suggest that their SDRs evolved independently. Thus, the ubiquity of trees 3 and 4 could result from the extreme degeneration of the Y in *M. picta* rather than from the true evolutionary history. It is possible to use read depth to identify and remove hemizygous sites, but these filters are not always accurate, especially with the very small sample sizes available to this study.

Further analysis suggests, however, that hemizygosity is unlikely to be responsible for all the SNPs associated with trees 3 and 4. Tree 3, in which the derived allele is unique to the X and Y chromosomes of *M. picta*, is 2.7 times more common than tree 4, in which the derived allele is unique to the X and Y chromosomes of *P. wingei*. The predominance of tree 3 is likely to be an artifact of degeneration in the Y of *M. picta*, as that tree will be erroneously inferred if a mutation that is unique to the X chromosome in *M. picta* occurs in a region deleted from its Y chromosome. Likewise, tree 4 can be erroneously inferred because of the same bioinformatic error if the true evolutionary history is tree 7. That evolutionary scenario is unlikely, however, since the origin of an X from a Y to our knowledge has never been observed. Tree 4 can also result from a mutation that is unique to the X chromosome of *P. wingei* in a region that is deleted from its Y chromosome. We found little evidence, however, of extensive Y degeneration in *P. wingei* based on the read depth ratio (Fig. 2). The large number of SNPs exhibiting tree 4 (about 10% of all SNPs in our dataset, including sites that are polymorphic within species) therefore support the hypothesis that the SDRs of *P. wingei* and *M. picta* originated independently.

The trees associated with sex chromosome turnover hypotheses (trees 5–8 in Fig. 5) are all very rare. Tree 5, seen at 42 SNPs, is consistent the origin of a new Y chromosome from the X in the ancestor of *P. wingei*. Alternatively, tree 5 could result from homoplasy if the same mutation independently occurred on one of the *M. picta* sex chromosomes and on a recombining region in *P. wingei* that later became part of the SDR.

In tree 7, which is seen at more than twice as many SNPs as tree 5, the X and Y of *P. wingei* both share a derived allele with the Y of *M. picta*. As noted above, this tree is consistent with the implausible hypothesis that the *wingei* X was derived from its Y. A more likely explanation is the homoplasy hypothesis described above for tree 5. The greater frequency of tree 7 relative to tree 5 could be due to higher mutation rates on the Y than on the X in *M. picta*, as observed in species with male-biased mutation (Wilson Sayres and Makova 2011). Male-biased mutation would also explain the greater frequency of tree 8 relative to tree 6.

Now consider how degeneration of the Y in *M. picta* might lead to the patterns seen in trees 5–8. Tree 5 can only be inferred at those rare sites in *picta* that are not hemizygous, while tree 6 can be erroneously inferred at hemizygous sites. This contrast could explain why tree 6 is much more common. The same logic could explain why tree 8 is much more common than tree 7. Finally, trees 5 and 6 could also result if the SDRs of the 2 species are homologous and a SNP that evolved in the ancestral X occurs in a region that was lost from one of the Y chromosomes. We believe this hypothesis is unlikely as that scenario is incompatible with trees 7 and 8, which are more common. In addition, the SNPs showing trees 5 and 6 occur throughout the chromosome rather than being localized in a region, as we expect for an ancestral SDR.

In sum, we find no evidence that supports the homology of the SDRs in *P. wingei* and *M. picta*. The patterns of allele sharing instead suggest that these SDRs originated independently after their lineages diverged. Three of the hypotheses shown in Fig. 4 are consistent with the data: an early turnover in the common ancestor of *P. reticulata* and *P. wingei* (as proposed by Charlesworth et al. 2021), a late turnover specific to the *P. reticulata* lineage, or SDRs originated by the independent suppression of recombination in the *M. picta* and *P. wingei* lineages. Charlesworth et al. (2021) reject this last hypothesis, arguing that degeneration of the Y in *M. picta* is too extensive to have occurred since the split with the *Poecilia* lineage. While that conclusion is plausible, we are not confident of it. A recent study by Metzger et al. (2021) concluded that the Y in *Micropoecilia* degenerated after that lineage split from the guppy’s ancestor. Furthermore, wildly variable rates of Y chromosome degeneration are known from sticklebacks (Sardell et al. 2021), suggesting that it may not be possible to make generalizations about rates of degeneration. Our conclusion is that the totality of existing evidence cannot decide decisively between these last 3 hypotheses.

### Reconciliation of past studies and best practices going forward

How can the many discrepancies between the conclusions from previous studies be reconciled, and how best can the field move forward? Some of the problems can be traced to semantic differences. We defer to the terminology for sex chromosomes defined by Bachtrog et al. (2011). The SDR, or SDR, is a segment of the sex chromosomes that includes the sex-determining factor and in which crossing over between the X and Y is completely suppressed. Within the SDR, there can be one or more strata, which are regions in which crossing over was suppressed at different times in the past (Lahn and Page 1999; Charlesworth 2018). The SDR may be as small as a nucleotide, as in the case of the fugu, or as large as most of the Y chromosome, as in mammals (Bachtrog et al. 2014). By this definition, it is a *non sequitur* to say there is a stratum on the guppy sex chromosomes in which there is rare crossover recombination (Darolti et al. 2020; Almeida et al. 2021). Again following Bachtrog et al. (2011), all of the X and Y that fall outside of the SDR make up the PARs, or PAR, regardless of whether the local recombination rate (measured as cM/Mb) is very high, as in mammals, or very low, as in some frogs (Bachtrog et al. 2014). By this definition, it is also a *non sequitur* to refer to only part of the recombining region as the PAR (Bergero et al. 2019). Authors are of course free to adopt noncanonical definitions, but in that case we urge them to give explicit definitions and use terms consistently.

In addition to these semantic issues, there are 3 substantive reasons why different studies have arrived at differing conclusions. First, some statistical approaches used to define the SDRs and PARs of the guppy and its relatives are problematic. Two nonrecombining strata were identified in *P. reticulata* and *P. wingei* using the criterion that molecular differences between males and females over a region of the sex chromosome fall outside the 95% confidence interval seen on autosomes (Wright et al. 2017; Darolti et al. 2019). By that standard, 5% of the entire genome is expected to be identified as nonrecombining sex chromosome strata, and indeed several regions of autosomes do meet that criterion (Supplementary Fig. 1 in Wright et al. 2017). Furthermore, the differences between males and females used to define nonrecombining strata in those studies are extremely small and likely not biologically meaningful [e.g. a male: female SNP density ratio differing much less than 1% from the autosomal average (Wright et al. 2017)]. The evidence reviewed above shows recombination is in fact occurring in the 2 strata. Thus, the bioinformatic strategy used by Wright et al. (2017), Darolti et al. (2019), and Almeida et al. (2021) appears to be underpowered to identify SDRs and strata.

Second, the results from linkage mapping experiments have been interpreted in different ways. It is difficult to draw conclusions from a failure to observe crossovers. Mapping experiments are underpowered to distinguish between partial and full linkage of loci to the male determining gene (Muyle et al. 2016; Wright et al. 2019). This limitation is particularly acute in species with extremely low recombination rates in males, such as the guppy. Conclusions about regions where the X and Y do not recombine based on linkage mapping should be treated with caution. Methods that define nonrecombining SDRs using gene trees are much more sensitive because they integrate genetic signatures of recombination over long periods of evolutionary time (e.g. Lahn and Page 1999; Zhou et al. 2014; Dixon et al. 2018; Sardell et al. 2021).

Third, sequencing studies have used different reference genomes and different strategies to map reads. Wright et al. (2017) used a de novo assembly of *P. reticulata* (N50 = 0.017 Mb) whose scaffolds were then oriented according to a guppy reference. Bergero et al. (2019) and Charlesworth et al. (2020a) mapped their DNA reads to the old *P. reticulata* reference (scaffold N50 = 31.4 Mb; Künstner et al. 2016). While this reference has the advantage of being from the species of interest, it is based entirely on scaffolded short-read sequences. Darolti et al. (2019; 2020) used one of the publicly available *Xiphophorus helleri* genomes (scaffold N50 = 29.4 Mb; Shen et al. 2016) to order scaffolds from their own de novo genome assemblies of *P. reticulata*. For most of the analyses in this study, we used the *X.maculatus* reference because it has the highest quality of any species in this family (Schartl et al. 2013). The current assembly (version 5.0) is based on long-read as well as short-read sequencing and optical mapping to produce the chromosome-level assembly (scaffold N50 = 31.5 Mb). To verify our findings in *X. maculatus*, we re-analyzed patterns of allele sharing on Chr 12 using the new *P. reticulata* reference genome (scaffold N50 = 32.8 Mb) that was recently published (Fraser et al. 2020), and obtained very similar results. Accordingly, we do not think that the use of different genome assemblies has contributed much to the conflicting conclusions reached by different research groups.

In sum, given the contrasting types of data and analyses that have been used, it is unsurprising that different research teams have come to conflicting conclusions. We believe that the major causes of these discrepancies are differences in the types of data, the analyses, and the interpretations.

## Conclusions

Five lines of evidence argue against the existence of a large nonrecombining SDR or strata on the sex chromosomes of the guppy, *P.reticulata*. In contrast, recombination between the X and Y in 2 closely related species (*P. wingei* and *M.picta*) is clearly suppressed. The degree of degeneration on the Y of *M. picta* is much more extensive. Patterns of allele sharing between the sex chromosomes of these 2 species strongly suggest that their SDRs are not homologous. The lesser degeneration of the SDR in *P. wingei* is likely because it originated more recently, either in a turnover event in which a new Y was derived from its X chromosome, or by suppression of recombination between the X and Y after the *P. wingei* and *M. picta* lineages diverged.

There is substantial potential for further progress in understanding the evolution of the sex chromosomes of guppies and their relatives. Many of the obstacles that have made study of the *Poecilia* and *Micropoecilia* so difficult might be resolved using whole-genome sequencing of experimentally phased sex chromosomes (see Sardell et al. 2021). That strategy can be complemented by long-read sequencing, which might eliminate many of the bioinformatic problems present in existing data. This hybrid approach would also enable the estimation of gene trees with greater confidence than is currently possible, leading to the robust solution of several issues that are still unresolved.

## Data availability

All data analyzed in this study came from the public repositories described in the *Materials and Methods*.

Supplemental material is available at *G3* online.

## Supplementary Material

jkab435_Supplementary_Data
