# Supplementary figures and images for: Evolution of the canonical sex chromosomes of the guppy and its relatives
Source: G3 (Bethesda). 2021 Dec 21;12(2):jkab435. doi: 10.1093/g3journal/jkab435 (PMC9335935; doi:10.1093/g3journal/jkab435)

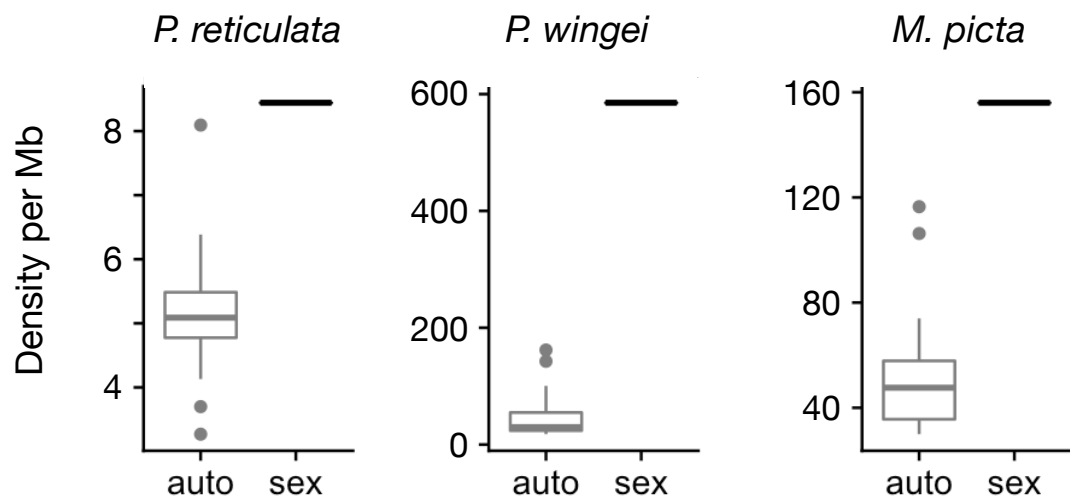

**Supplementary figure S1.** Densities of SDR-like SNPs on sex chromosomes and autosomes.

Supplement: jkab435_Supplementary_Data [file jkab435_supplementary_data.pdf]
